# Upregulation of Placental Vitamin D Receptor Expression in Gestational Diabetes Is Not Directly Related to Vitamin D Concentration

**DOI:** 10.3390/biology14091300

**Published:** 2025-09-20

**Authors:** Marek Walkowiak, Małgorzata Jamka, Mateusz de Mezer, Jakub Żurawski, Paweł Gutaj, Ewa Wender-Ożegowska

**Affiliations:** 1 Department of Reproduction, Chair of Feto-Maternal Medicine, Poznan University of Medical Sciences, Polna Str. 33, 60-535 Poznan, Poland; pawelgutaj@ump.edu.pl (P.G.); e.wenderozegowska@ump.edu.pl (E.W.-O.); 2 Doctoral School, Poznan University of Medical Sciences, Bukowska Street 70, 60-812 Poznan, Poland; 3 Department of Pediatric Gastroenterology and Metabolic Diseases, Poznan University of Medical Sciences, Szpitalna Street 27/33, 60-572 Poznan, Poland; 4 Department of Immunobiology, Poznan University of Medical Sciences, Rokietnicka Street 8, 60-806 Poznan, Poland; mdemezer@ump.edu.pl (M.d.M.); zurawski@ump.edu.pl (J.Ż.)

**Keywords:** gestational diabetes, vitamin D, vitamin D receptor, placenta, diabetes mellitus

## Abstract

Vitamin D plays a key role in pregnancy beyond calcium–phosphate regulation, modulating immune responses and glucose metabolism via the vitamin D receptor (VDR). Placental expression may be altered in gestational diabetes mellitus (GDM). This study aimed to assess placental VDR expression in GDM and evaluate its association with maternal vitamin D levels and clinical parameters. VDR expression in the placental tissue of 53 women with GDM and 26 healthy controls was assessed semi-quantitatively by immunohistochemistry. Maternal serum 25-hydroxyvitamin D (25(OH)D) levels, body mass index, weight gain, neonatal outcomes, and other variables were evaluated. Univariate and multivariate linear regression analyses were performed. VDR expression was significantly higher in the GDM group compared to controls (*p* = 0.0297 for mean, *p* = 0.0378 for median). No significant differences were observed in serum 25(OH)D concentrations between groups. Stepwise regression revealed that diabetes was the only independent predictor of VDR expression. Within the GDM subgroup, VDR expression was not associated with any clinical parameters, including maternal vitamin D status. Upregulated placental VDR in GDM may represent an adaptive response to metabolic stress. These findings suggest complex regulation of vitamin D signalling in diabetic pregnancies, warranting further investigation.

## Simple Summary

Vitamin D plays an important role in pregnancy, affecting the immune system and metabolism. It seems to be involved in the pathophysiology of gestational diabetes, although the exact mechanisms are not yet fully understood. This study aimed to examine the expression of the vitamin D receptor in the placenta of patients with gestational diabetes mellitus and healthy women. Placental expression of the vitamin D receptor was higher in women with diabetes compared with controls, while serum vitamin D levels were similar in both groups. Diabetes is associated with expression of the receptor independently of a number of clinical characteristics. The observed increase in the placental expression of the vitamin D receptor in gestational diabetes mellitus may be an adaptive response to metabolic stress, unrelated to maternal vitamin D levels. These findings warrant further investigation.

## 1. Introduction

Beyond regulating calcium metabolism and immune response, during pregnancy, vitamin D plays a vital role in maintaining insulin sensitivity and reducing inflammation. Vitamin D acts through the vitamin D receptor (VDR) to regulate glucose metabolism and inhibit several cytokines [1]. Previous studies found an association between low vitamin D concentration (<20–30 ng/mL) and elevated gestational diabetes mellitus (GDM) risk (relative risk (RR) = 1.5–3) [2,3]. The risk of GDM appears to be elevated in higher latitudes during winter [4]. Such observed annual fluctuations could not be attributed to some unspecified seasonality, as a Cochrane meta-analysis has suggested that vitamin D supplementation reduces the risk of GDM (RR = 0.51, 95% confidence interval (CI): 0.27–0.97) [5]. High-dose supplementation (e.g., 5000 IU/day) appears to be most effective with high doses before the 20th week of gestation [6,7]. However, some trials found no GDM risk reduction even with high-dose, early supplementation [8]. These mixed outcomes, influenced by body mass index (BMI) and VDR polymorphisms, suggest VDR’s metabolic role may outweigh 25-hydroxyvitamin D (25(OH)D) levels in GDM [9].

Few studies on VDR expression during GDM hint at a more complex relationship. In GDM placentas, VDR protein and mRNA are 1.8-fold upregulated in extravillous trophoblasts and 5.8-fold in foetoplacental endothelial cells, potentially driven by hyperglycaemia or inflammation, though low maternal 25(OH)D concentrations may be the trigger [10]. Serum VDR levels are also elevated in GDM women, correlating with insulin resistance despite adequate 25(OH)D levels, suggesting a systemic metabolic response [11]. In contrast, VDR and peroxisome proliferator-activated receptor γ mRNA are upregulated in the adipose tissue of GDM women, particularly in overweight or obese cases, indicating that obesity may amplify VDR expression [12]. VDR levels are higher in fat tissue and certain immune cells in type 2 diabetes, but lower in skeletal muscle because high blood glucose changes gene expression [7]. These tissue-specific patterns highlight that hyperglycaemia does not uniformly increase VDR, underscoring the need for placental studies to clarify its role in GDM’s unique metabolic environment [12]. Therefore, this study was designed to investigate the role of GDM in placental VDR expression. Given the paucity of research on placental VDR in GDM, this work addresses a critical gap by examining how VDR integrates vitamin D signalling, hyperglycaemia, and inflammation to influence obstetric outcomes [11]. VDR polymorphisms affect the risk of GDM, but may have a diverse influence depending on geographical regions [13]. By measuring VDR’s placental function in GDM, this study aims to clarify its contribution to maternal–foetal health, paving the way for targeted research into metabolic and genetic mechanisms.

## 2. Materials and Methods

### 2.1. Ethics

This prospective cross-sectional study was conducted from April 2022 to June 2024 at the Gynaecological–Obstetrical University Hospital (GOUH) of the Poznan University of Medical Sciences (PUMS) according to the guidelines of the Declaration of Helsinki. It was approved by the Bioethics Committee of the PUMS (459/20), and all participants provided informed consent. This study was supported by the internal grant for doctoral students from the School of Doctoral Studies at the PUMS (Grant no. SDUM-GB19/03/21).

### 2.2. Study Population

The inclusion criteria were age > 18 years, singleton pregnancy, gestational age > 35 weeks, Caucasian origin, and consent for delivery at the GOUH. The study group had GDM, whereas the control group had a pre-pregnancy BMI of 18.5–25 kg/m^2^ and negative screening for GDM. The exclusion criteria comprised foetal malformations; preterm delivery before the end of 34 weeks of gestation; premature ablation of the placenta; any other factors which, in the opinion of the investigator, may hinder the conduct of the study; and type 1 or type 2 diabetes diagnosed before pregnancy.

Data, including maternal age and height, pre-pregnancy and perinatal body weight and BMI, weight gain during pregnancy, gestational age at birth, neonatal sex and weight (expressed in grams, as well as in percentages and Z-scores) [14], maternal parity, and placental weight, were collected for analysis.

GDM was diagnosed according to the International Association of Diabetes and Pregnancy Study Groups criteria [15] and Polish standards [16,17]. Gestational age was verified and confirmed by ultrasound in the first trimester.

### 2.3. Biochemical and Histological Assessment

Whole blood from venous access (volume not exceeding 7.5 mL), as well as placental fragments, was collected perinatally from all participants. Serum vitamin D concentrations and glycated haemoglobin (HbA1c) levels were measured in whole blood, and VDR expression was quantified in the placental fragments.

Vitamin D levels were measured using an immunoassay method on the Alinity i analyser in a commercial laboratory (Diagnostyka SA, Poznan, Poland). Vitamin D concentrations were evaluated according to the Polish guidelines: 0–20 ng/mL—deficiency; 20–30 ng/mL—suboptimal level; >30–50 ng/mL—optimal level; >50–100 ng/mL—high level; >100 ng/mL—potentially toxic level [18].

Placental tissue was obtained from all subjects for histological assessment using a rabbit polyclonal antibody to VDR (Biorbyt orb214726, Cambridge, UK) and non-immune immunoglobulin G (IgG) as a negative control. The spleen was a positive control for immunohistochemical staining. Briefly, 5 mm thick tissue sections were dewaxed with xylene and then dehydrated with a series of alcohols. Antigens were exposed at 96 °C for 20 min in citrate buffer, pH 6.0 (H-3300), and endogenous peroxidase activity was quenched in BLOXALL blocking solution for 10 min.

Nonspecific bonds were blocked in 2.5% normal horse serum from the ImmPRESS^®^ Horse Anti-Rabbit IgG PLUS Polymer Kit Peroxidase reagent kit (Vector Laboratories, Newark, CA, USA, MP-7801) for 20 min. Then, the excess serum was removed from the sections. In the next step, they were incubated overnight at 4 °C with antibodies against the analysed proteins. The slides were rinsed in PBS buffer for 5 min and incubated for 30 min with the ImmPRESS reagent. The slides were then rinsed twice for 5 min in PBS buffer before incubation in ImmPACT DAB EqV solution until the desired colour was achieved. The sections were rinsed twice for 5 min in PBS and stained with haematoxylin. Finally, the sections were dehydrated in a series of alcohols and xylene and covered with cover slips.

Ten images of the field of view were taken at 400× using an Olympus Grundium Ocus 40 microscopic scanner (Olympus, Tokyo, Japan). A semi-quantitative assessment of the immunohistochemical reactions was conducted based on the photographic documentation, utilising the commercial Olympus cellSens dimensional program. This programme performs phase analysis of the stained specimen, which consists of automatic detection of objects due to their colour (brown chromogen DAB-3.3). Thresholds were introduced according to which the software automatically classified the data. During the preparation phase, cell counts and the surface area of immunohistochemical reactions were evaluated and expressed in mm^2^, and then the results were automatically exported to MS Excel sheets for further statistical analysis.

All immunohistochemical analyses were performed at the Department of Immunobiology, PUMS [19]. Figures with high and low VDR expression have been presented in Supplementary Files (Figures S1 and S2).

### 2.4. Sample Size Calculation

The G*Power 3.1 software (University of Kiel, Kiel, Germany) was used to calculate the minimum required sample size based on the following assumptions: type I error probability (α) = 0.05, type II error probability (β) = 0.05, and an anticipated dropout rate = 20% using vitamin D levels reported by Vijay et al. [20]. A minimum of 20 participants in each group should be recruited for the study.

### 2.5. Statistical Analysis

Statistical analysis was performed using the Statistica 13.0 software (TIBCO Software Inc., Palo Alto, CA, USA). The level of significance was set at *p* < 0.05, and a CI of 95% was applied. The normality of the data distribution was assessed by the Shapiro–Wilk test, with Levene’s test used to assess the equality of variances. Numerical data are presented as medians and 1st–3rd quartiles (Q1–Q3) and Boolean as a number [percentage (%)]. Unpaired comparisons between two groups were conducted using the Mann–Whitney U test. The differences between women with and without GDM were examined using analysis of covariance, adjusted for vitamin D levels. The relationships between categorical variables were examined using Fisher’s exact test. Univariable and multivariable linear regression analyses were performed to assess the independent association between placental VDR median expression and selected variables (age, weight gain during pregnancy, pre-pregnancy BMI, gestational age at birth, newborn weight, maternal vitamin D levels, and carbohydrate metabolism disorders) separately in the total population and pregnant women with diabetes. In addition, backwards and forward stepwise regression analyses were also conducted.

## 3. Results

Fifty-three patients with GDM and 26 healthy controls were recruited (Table 1). GDM patients were significantly older, had a higher body weight and BMI (both before pregnancy and before delivery). Vitamin D concentrations in mothers and parameters of their newborns, beyond gestational age, did not differ between groups. The difference in placental weight did not reach the level of significance (*p* = 0.0541). VDR expression was significantly higher in GDM (*p* = 0.0297 and *p* = 0.0378 for mean and median, respectively) (Table 1, Figure S3). Adjusting the results for vitamin D concentrations did not change the findings (Table 1).

Univariate and multivariate linear regression analyses revealed that diabetes and gestational age were significantly associated with VDR expression (median), but none of the parameters were independent predictors (Table 2 and Table 3). 

Diabetes was the only predictor of placental VDR expression according to backwards and forward stepwise regression (Table 4).

Univariate and multivariate linear regression analyses assessing the relationship of selected variables with VDR (median) in the GDM group indicated that none of the parameters were associated with VDR expression (Table 5 and Table 6).

## 4. Discussion

The present study proved that placental VDR expression was significantly higher in GDM than in healthy controls. However, none of the epidemiological and clinical parameters were found to be independent risk factors responsible for the higher VDR expression.

The observed upregulation of VDR expression in GDM placentas in the present study, combined with no differences in vitamin D_3_ levels compared to healthy women, challenges previously reported trends and offers a new perspective on the role of vitamin D signalling in GDM pathophysiology. Cho et al. [21] documented that placental VDR protein and mRNA expression do not differ between patients with GDM and healthy women, whereas Knabl et al. [10] reported upregulated VDR protein and mRNA levels. The findings were more pronounced in extravillous than in villous trophoblasts. Interestingly, high calcitriol doses decreased, whereas low concentrations increased upregulation in the additional experiment carried out using the trophoblast tumour BeWo cell line. Subsequently, VDR expression was linked to reduced vitamin D_3_ concentrations [10]. Our findings suggest that placental VDR expression in GDM may be more complex, being dynamically regulated and potentially influenced by many context-specific factors, not excluding gestational age at delivery, maternal vitamin D status, BMI, glycaemic control, or pharmacologic treatment.

It is of note that differences in study populations, sample sizes, quantification methods (e.g., quantitative polymerase chain reaction (qPCR) vs. immunohistochemistry), and placental sampling sites may contribute to divergent findings across studies. Our results underscore the complexity of vitamin VDR signalling in GDM and highlight the need for integrated analyses of maternal vitamin D status, placental gene expression, and downstream functional pathways.

One possible explanation for the increased VDR expression is a compensatory placental response to altered maternal–foetal vitamin D metabolism or hyperglycaemia-induced inflammation [22]. Vitamin D signalling has well-documented immunomodulatory properties, including the suppression of pro-inflammatory cytokines and enhancement of anti-inflammatory pathways [9,23]. Given the chronic low-grade inflammation and oxidative stress associated with GDM [24], it is plausible that the placenta upregulates VDR as a protective mechanism to mitigate local inflammatory responses and maintain placental function.

Additionally, upregulated VDR expression may influence downstream gene networks involved in glucose metabolism, insulin signalling, and lipid transport, potentially impacting foetal growth and development [1]. For example, VDR is known to regulate insulin receptor gene expression [7] and may also modulate placental lipid handling and nutrient transport [25].

Epigenetic regulation may also play a role in the observed findings. Novakovic et al. [26] reported tissue-specific methylation patterns of the *CYP24A1* gene in the placenta, which may impact active vitamin D availability and VDR signalling. It is conceivable that epigenetic alterations in the VDR gene or VDR-regulated targets could drive an increase in VDR expression in GDM.

The relationship between VDR expression and GDM risk is complex and may extend beyond basic genetic polymorphisms with more complicated interactions. There are four key polymorphisms within the *VDR* gene (NCBI Gene ID: 7421): FokI (rs2228570), which introduces an early start codon shortening the protein; the intronic variants ApaI (rs7975232) and BsmI (rs1544410); and the synonymous variant TaqI (rs731236) [27]. Although published VDR gene studies meta-analyses are based on relatively small samples, they suggest varied impacts among populations: ApaI appears to be a stronger GDM risk factor in East Asians than in Europeans, while BsmI increases risk only in East Asians, Fokl primarily among Europeans, and TaqI lacks an unambiguous impact [28]. These meta-analyses support that ApaI affects all races, as does BsmI, except potentially in South American populations, while TaqI and FokI show reversed impacts across races [28,29]. This ethnic discrepancy is plausible, as other meta-analyses of genetic GDM predictors have noted a similar paradox: variants like TCF7L2 (rs7903146) and PPARG (rs1801282) increase risk in East Asians but lack impact in Europeans [30], with a subsequent Polish study failing to detect any TCF7L2 effect [31]. While FokI, ApaI, BsmI, and TaqI variants were linked in a study of Saudi women with elevated GDM risk, ApaI was the only variant not associated with decreased serum vitamin D levels, suggesting a more complex metabolic pathway [32].

This study has several limitations that must be acknowledged. Although immunohistochemistry was employed for localisation and semi-quantitative assessment, more sensitive molecular techniques such as reverse transcription (RT)-qPCR or Western blotting might provide additional insights into VDR regulation. Second, obesity is one of the primary risk factors for GDM, and as such, its prevalence is significantly higher in this group compared to the general population. In designing the study, we aimed for the control group to be free of additional confounding factors, including obesity, to provide a more reliable reference point. Given the well-established association between obesity and GDM, we deliberately included patients both with normal and abnormal body mass in the study group. Third, the cross-sectional nature of the sampling precludes conclusions about temporal changes in VDR expression throughout pregnancy. Finally, the relatively small sample size may limit the assessment of interactions with epidemiological and clinical factors. Neonatal health status is a good example of a multifactorial outcome, which is closely influenced by the mode and course of delivery, gestational age, maternal age, and numerous other variables. At the same time, identifying objective and standardized indicators for its assessment remains challenging. Therefore, further studies are needed, including larger groups.

Despite these limitations, our findings provide novel evidence regarding vitamin D signalling in the placentas of women with GDM, suggesting a potential pathway linking maternal metabolic dysregulation with altered placental function. Future studies should investigate the molecular regulators of placental VDR expression, functional consequences on placental and foetal physiology, and evaluate whether targeted interventions could restore normal vitamin D signalling in diabetic pregnancies.

## 5. Conclusions

This study demonstrates that placental VDR expression is significantly increased in pregnancies complicated by gestational diabetes mellitus, independent of maternal serum vitamin D levels. These results suggest that VDR upregulation may serve as an adaptive response to metabolic or inflammatory stress rather than being solely driven by vitamin D availability.

### 5.1. Key Conclusions

- VDR expression is elevated in the placentas of women with GDM, regardless of maternal 25(OH)D levels.
- Diabetes status, rather than BMI, gestational age, placental weight, or vitamin D levels, is the only variable independently associated with increased VDR expression.
- The findings imply a compensatory placental mechanism potentially aimed at counteracting the adverse effects of maternal metabolic dysregulation.

### 5.2. Future Directions

1. Future studies should incorporate more sensitive molecular techniques, such as RT-qPCR or Western blotting, to better quantify VDR expression and its regulatory mechanisms.
2. Investigation of VDR gene methylation and other epigenetic modifications may help explain interindividual expression differences.
3. Further research is needed to elucidate how increased placental VDR expression impacts nutrient transport, placental inflammation, and foetal development.
4. Modulating placental VDR signalling could represent a novel therapeutic target for mitigating adverse outcomes in pregnancies affected by GDM.

## Figures and Tables

**Table 1:** Comparison of the studied women with and without diabetes.

		Median (Q1–Q3)		*p* ^5^	*p* ^7^
		No Diabetes (*n* = 26)	GDM (*n* = 53)		
Age [years]		30 (26–32)	33 (29–37)	0.0030	0.0054
Weight before pregnancy [kg]		61.5 (53.0–67.0)	79.0 (64.0–95.0)	0.0001	0.0001
Maternal perinatal weight [kg]		75.5 (63.0–82.0)	87.0 (73.0–108.0)	0.0049	0.0080
Weight gain during pregnancy [kg]		13.0 (11.0–18.0)	9.0 (5.0–13.0)	0.0011	0.0075
Height [cm]		167 (163–175)	165 (160–170)	0.1535	0.1256
Pre-pregnancy BMI [kg/m^2^]		21.26 (19.37–23.62)	26.79 (23.87–33.91)	<0.0001	<0.0001
Gestational age [week]		39 (38–40)	38 (38–39)	0.0003	0.0001
Number of pregnancies [*n*]		1 (1–3)	3 (2–4)	0.0004	0.0006
Number of births [*n*]		0 (0–1)	1 (0–2)	0.0053	0.0011
Newborn weight [g]		3532.5 (3250.0–3650.0)	3440.0 (3040.0–3960.0)	0.7702	0.4623
Newborn weight [percentile]		58.5 (28–72)	59 (25–92)	0.6018	0.6036
Newborn weight [Z-score]		0.21 (−0.59–0.57)	0.24 (−0.66–1.4)	0.5874	0.7851
Placental weight [g]		600 (550–650) ^1^	640 (590–750) ^2^	0.0541	0.0516
Maternal HbA1c [%]		-	5.3 (5.1–5.6) ^3^	-	-
Placental VDR mean		5852 (4900–7048) ^1^	8087 (5324–13869)	0.0297	0.0263
Placental VDR median		5813 (4923–7024) ^1^	8110 (5089–13712)	0.0378	0.0319
Maternal 25(OH)D [ng/mL]		32.95 (28.10–38.90)	28.65 (16.15–39.05) ^4^	0.1682	-
		***n* [%]**		***p* ^6^**	
Sex of neonate	Male	11 (42%)	30 (57%)	0.3380	
	Female	15 (58%)	23 (43%)		

^1^ *n* = 25; ^2^ *n* = 51; ^3^ *n* = 46; ^4^ *n* = 52; ^5^ Mann–Whitney U test; ^6^ Fisher’s exact test; ^7^ adjusted for maternal 25(OH)D [ng/mL]. BMI—body mass index; GDM—gestational diabetes mellitus; HbA1c—glycated haemoglobin; VDR—vitamin D receptor; 25(OH)D—25-hydroxyvitamin D.

**Table 2:** Univariate regression analysis assessing the relationship between placental VDR median and selected variables in the total population.

VDR Median	Parameter	SE	t	*p*	95% CI	β	SE β	95% CI for β
Age [year]	34.16	115.96	0.2946	0.7691	−196.79–265.12	0.03	0.11	−0.19–0.26
Weight gain during pregnancy [kg]	−43.18	91.57	−0.4716	0.6386	−225.56–139.19	−0.05	0.11	−0.28–0.17
Pre-pregnancy BMI [kg/m^2^]	84.15	86.32	0.9749	0.3327	−87.76–256.07	0.11	0.11	−0.12–0.34
Gestational age [week]	−1164.03	562.96	−2.0677	0.0421	−2285.26–−42.80	−0.23	0.11	−0.45–−0.01
Newborn weight [g]	−1.38	1.12	−1.2371	0.2199	−3.61–0.84	−0.14	0.11	−0.37–0.08
Maternal 25(OH)D [ng/mL]	14.05	40.31	0.3486	0.7284	−66.25–94.35	0.04	0.12	−0.19–0.27
Diabetes [No]	−1384.50	644.75	−2.1473	0.0350	−2668.63–−100.36	−0.24	0.11	−0.46–−0.02

BMI—body mass index; CI—confidence interval; SE—standard error; VDR—vitamin D receptor; 25(OH)D—25-hydroxyvitamin D.

**Table 3:** Multivariate linear regression analysis assessing the relationship between placental VDR median and selected variables in the total population. All variables from the univariate analysis were included in the model.

VDR Median	Parameter	SE	t	*p*	95% CI	β	SE β	95% CI for β
Age [year]	−38.80	124.15	−0.3125	0.7556	−286.48–208.88	−0.04	0.12	−0.28–0.21
Weight gain during pregnancy [kg]	69.49	103.67	0.6703	0.5049	−137.32–276.30	0.09	0.13	−0.17–0.35
Pre-pregnancy BMI [kg/m^2^]	44.51	106.42	0.4182	0.6771	−167.80–256.82	0.06	0.14	−0.22–0.34
Gestational age [week]	−693.04	674.99	−1.0268	0.3081	−2039.60–653.52	−0.14	0.13	−0.40–0.13
Newborn weight [g]	−1.25	1.39	−0.9009	0.3708	−4.02–1.52	−0.13	0.14	−0.41–0.15
Maternal 25(OH)D [ng/mL]	7.00	42.40	0.1651	0.8693	−77.59–91.59	0.02	0.12	−0.22–0.26
Diabetes [No]	−1100.38	877.58	−1.2539	0.2141	−2851.11–650.34	−0.19	0.15	−0.49–0.11

BMI—body mass index; CI—confidence interval; SE—standard error; VDR—vitamin D receptor; 25(OH)D—25-hydroxyvitamin D.

**Table 4:** Backwards/forward stepwise regression assessing the relationship between placental VDR median and selected variables in the total population.

VDR Median	Parameter	SE	t	*p*	95% CI	β
Diabetes [No]	−1399.24	650.67	−2.1504	0.0347	−2695.44–−103.03	−0.11

CI—confidence interval; SE—standard error; VDR—vitamin D receptor.

**Table 5:** Univariate regression analysis assessing the relationship between placental VDR median and selected variables in pregnant women with diabetes.

VDR Median	Parameter	SE	t	*p*	95% CI	β	SE β	95% CI for β
Age [year]	−15.93	144.32	−0.1104	0.9125	−305.67–273.81	−0.02	0.14	−0.30–0.26
Weight gain during pregnancy [kg]	54.30	109.49	0.4959	0.6221	−165.51–274.11	0.07	0.14	−0.21–0.35
Pre-pregnancy BMI [kg/m^2^]	17.38	106.91	0.1625	0.8715	−197.25–232.01	0.02	0.14	−0.26–0.30
Gestational age [week]	−868.83	891.07	−0.9750	0.3341	−2657.74–920.07	−0.14	0.14	−0.41–0.14
Newborn weight [g]	−0.26	1.34	−0.1914	0.8490	−2.94–2.43	−0.03	0.14	−0.31–0.25
Maternal HbA1c [%]	1001.42	1589.82	0.6299	0.5320	−2202.65–4205.49	0.09	0.15	−0.21–0.40
Maternal 25(OH)D [ng/mL]	35.47	46.44	0.7638	0.4486	−57.80–128.74	0.11	0.14	−0.17–0.39

BMI—body mass index; CI—confidence interval; HbA1c—glycated haemoglobin; SE—standard error; VDR—vitamin D receptor; 25(OH)D—25-hydroxyvitamin D.

**Table 6:** Multivariate linear regression analysis assessing the relationship between placental VDR median and selected variables in pregnant women with diabetes. All variables from the univariate analysis were included in the model.

VDR Median	Parameter	SE	t	*p*	95% CI	β	SE β	95% CI for β
Age [year]	39.84	164.43	0.2423	0.8099	−293.33–373.01	0.04	0.16	−0.29–0.37
Weight gain during pregnancy [kg]	91.56	136.14	0.6726	0.5054	−184.29–367.42	0.12	0.17	−0.24–0.47
Pre-pregnancy BMI [kg/m^2^]	61.00	145.53	0.4192	0.6775	−233.86–355.87	0.08	0.19	−0.31–0.47
Gestational age [week]	−1750.24	1198.96	−1.4598	0.1528	−4179.57–679.09	−0.23	0.16	−0.56–0.09
Newborn weight [g]	−1.67	1.90	−0.8806	0.3842	−5.52–2.18	−0.17	0.20	−0.57–0.23
Maternal HbA1C [%]	1441.74	2183.28	0.6604	0.5131	−2982.01–5865.48	0.14	0.21	−0.28–0.55
Maternal 25(OH)D [ng/mL]	13.47	56.57	0.2380	0.8132	−101.16–128.09	0.04	0.17	−0.30–0.38

BMI—body mass index; CI—confidence interval; HbA1c—glycated haemoglobin; SE—standard error; VDR—vitamin D receptor; 25(OH)D—25-hydroxyvitamin D.

## Data Availability

The data presented in this study are available upon request from the corresponding authors.

## Supplementary Materials

The following supporting information can be downloaded at: https://www.mdpi.com/article/10.3390/biology14091300/s1, Figure S1: High placental vitamin D receptor expression; Figure S2: Low placental vitamin D receptor expression. Figure S3: Serum Vitamin D concentration vs placental Vitamin D Receptor (VDR) expression.

## Abbreviations

The following abbreviations are used in this manuscript:

VDR	Vitamin D receptor
GDM	Gestational diabetes mellitus
25(OH)D	25-hydroxyvitamin D
RR	Relative risk
CI	Confidence interval
BMI	Body mass index
GOUH	Gynaecological–obstetrical university hospital
PUMS	Poznan University of Medical Sciences
HbA1c	Glycated haemoglobin
IgG	Immunoglobulin G
Q1–Q3	1st–3rd quartiles
SE	Standard error
qPCR	Quantitative polymerase chain reaction
RT	Reverse transcription
