# Supplementary material for: Upregulation of Placental Vitamin D Receptor Expression in Gestational Diabetes Is Not Directly Related to Vitamin D Concentration
Source: Biology (Basel). 2025 Sep 20;14(9):1300. doi: 10.3390/biology14091300 (PMC12467713; doi:10.3390/biology14091300)

Supplementary figures description

Figure S1 High placental vitamin D receptor expression
Figure S2 Low placental vitamin D receptor expression

Figure S1

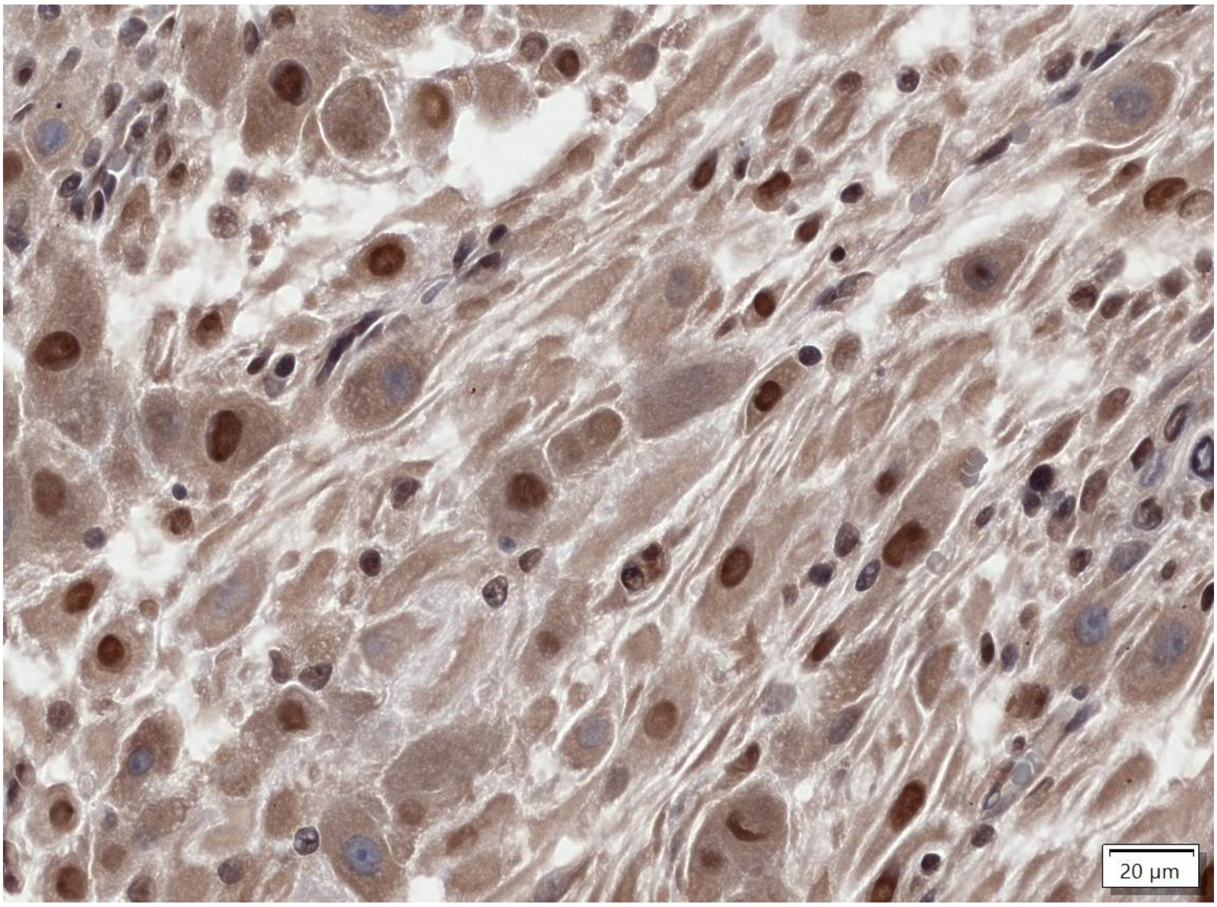

Figure S2

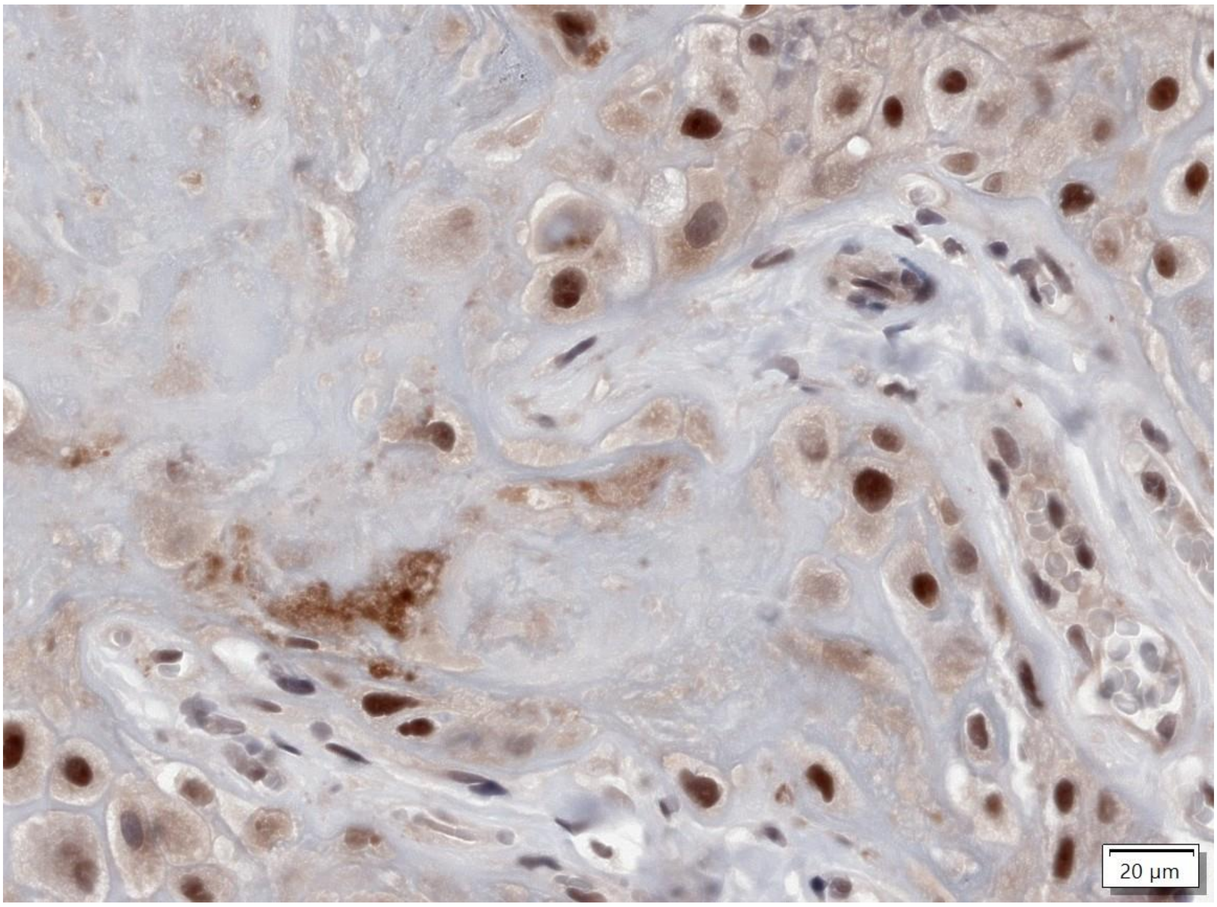

Supplement: Supplementary file 1 [file biology-14-01300-s001.zip › biology-3847822-S1 S2.pdf]
