# Supplementary figures and images for: Upregulation of Placental Vitamin D Receptor Expression in Gestational Diabetes Is Not Directly Related to Vitamin D Concentration
Source: Biology (Basel). 2025 Sep 20;14(9):1300. doi: 10.3390/biology14091300 (PMC12467713; doi:10.3390/biology14091300)

Serum vitamin D concentration vs placental vitamin D receptor expression (VDR)  
(Pearson  $r = 0.069$ ,  $p = 0.5525$ )

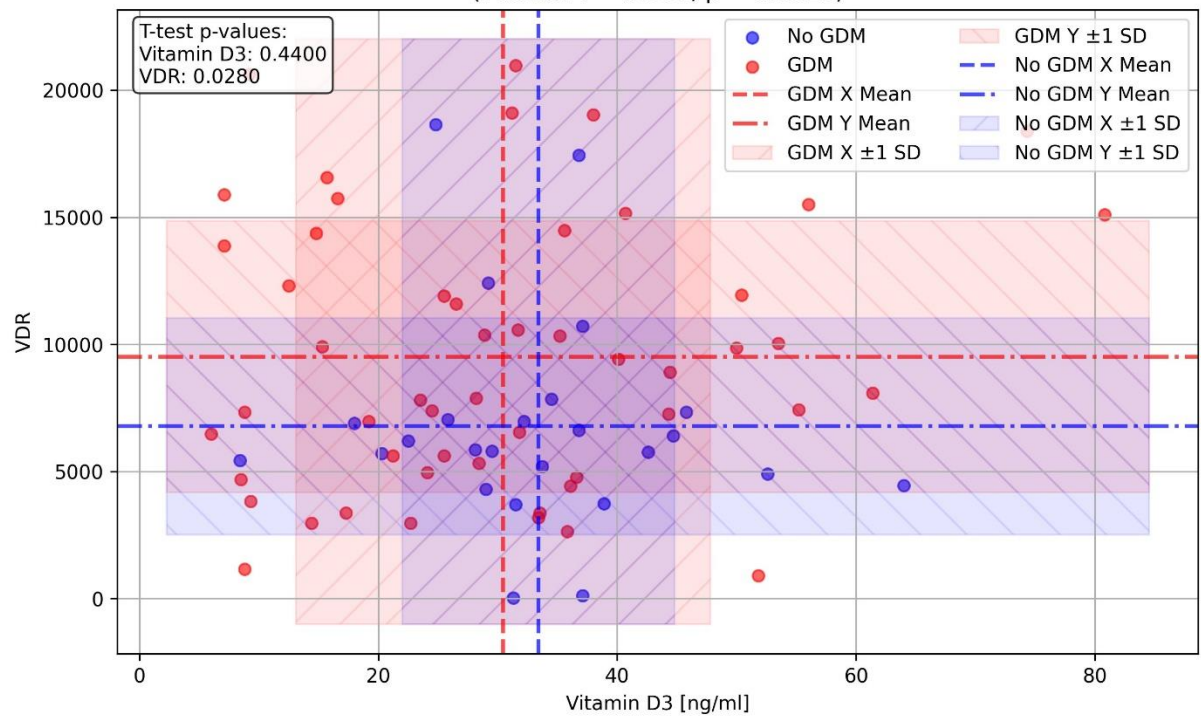

Supplement: Supplementary file 1 [file biology-14-01300-s001.zip › biology-3847822-S3.pdf]
